# Cytokines Driven Anti-Inflammatory and Anti-Psoriasis Like Efficacies of Nutraceutical Sea Buckthorn (*Hippophae rhamnoides*) Oil

**DOI:** 10.3389/fphar.2019.01186

**Published:** 2019-10-11

**Authors:** Acharya Balkrishna, Sachin Shridhar Sakat, Kheemraj Joshi, Kamal Joshi, Vinay Sharma, Ravikant Ranjan, Kunal Bhattacharya, Anurag Varshney

**Affiliations:** ^1^Drug Discovery and Development Division, Patanjali Research Institute, Haridwar, India; ^2^Department of Allied Sciences, University of Patanjali, Patanjali YogPeeth, Haridwar, India

**Keywords:** sea buckthorn oil, nutraceutical, anti-inflammatory activity, paw edema, psoriasis, 12-O-tetradecanoyl phorbol-13-acetate, THP-1, cytokines

## Abstract

Psoriasis is a chronic inflammatory skin disease characterized by circumscribed, red, thickened plaques with overlying silvery white scales. It is associated with the release of pro-inflammatory mediators that lead to the development of edema and distress. Here we show the anti-inflammatory and anti-psoriatic efficacies of a neutraceutical sea buckthorn oil (SBKT) derived from the fruit pulp of *Hippophae rhamnoides*. Chemical analysis of the SBKT showed the presence of 16 major saturated, mono-, and polyunsaturated fatty acids components, imparting significant nutritional values. Efficacy of the SBKT in modulating psoriasis and associated inflammation was first tested *in vitro* using human monocytic (THP-1) cells. SBKT induced cytotoxicity at a dose of ≥25 µl/ml. Treatment of the lipopolysaccharide-stimulated THP-1 cells with SBKT subdued the enhanced release of intracellular reactive nitrogen species and expression of NF-κB protein, in a concentration-dependent manner. This was accompanied by a reduction in the release of downstream pro-inflammatory cytokines: Interleukin-1ß and interleukin-6. Tumor necrosis factor-α released in the stimulated THP-1 cells were also inhibited by SBKT dose of 5 µl/ml. *In vivo* oral and topical treatment with SBKT in the Carrageenan-stimulated paw edema model, showed a significant decrease in paw volume and edema. In the 12-O tetradecanoyl phorbol 13-acetate (TPA) stimulated CD-1 mice psoriasis-like model, concurrent oral and tropical SBKT treatments substantially reduced ear edema and ear biopsy weights. Histopathologically, significant reduction in ear epidermal thickness and skin lesion scores was observed in the SBKT-treated animals. In conclusion, SBKT showed anti-inflammatory and anti-psoriasis-like efficacies in healing chemical-induced inflammation and psoriasis. The possible mode of action of SBKT was found through inhibition of reactive nitrogen species, and downregulation of NF-κB protein and pro-inflammatory cytokines. Thus, the present data suggest that Sea buckthorn oil can be used as an anti-inflammatory and anti-psoriatic nutraceutical.

## Introduction

Inflammation is induced as a response by the immune system to stimulations by invading foreign pathogens or by endogenous signals originating from damaged cells. While the primary function for pro-inflammatory cells is to counter the inducer and perform damage repair, sustained and unchecked inflammation can lead to the development of pathologies and induction of chronic diseases. Psoriasis is one such chronic inflammatory disease of skin and joints that affects 2–3% of the population of the world at the age of <40 years (Lebwohl, 2003; Wagner et al., 2010; World Health Organization, 2016). General symptoms of the psoriasis are circumscribed, red, and thickened plaques with an overlying silver-white scale(s) inducing itching, burning, and irritation.

Pathological signatures for psoriasis development include enhanced keratinocyte proliferation in the basal and suprabasal epidermal regions, the diminished thickness of the stratum granulosum, hyperkeratosis, and parakeratosis (Gudjonsson and Elder, 2008). Critical inflammatory cells such as macrophages, dendritic cells, neutrophils, lymphocytes, and T helper (T_H_) cells infiltrate the psoriasis lesions, and along with the stressed keratinocytes produce reactive oxygen and nitrogen species, and pro-inflammatory cytokines and chemokines (Liu, 2005; Richardson and Gelfand, 2008; Wagner et al., 2010). Currently, there are no permanent treatments for psoriasis, and anti-inflammatory drugs are topically applied during the flaring of the disease.

The term “nutraceutical” has been derived from the combination of “nutrition” and “pharmaceutical” to describe a nutritional product with pharmaceutical effects (Das et al., 2012). Nutraceuticals application includes a range of therapeutic areas like skin diseases, sleeping disorders, osteoporosis, metabolic disorders, etc. Sea buckthorn [*Hippophae rhamnoides* L.; sea buckthorn oil (SBKT)] belongs to the plant family Elaeagnaceae. It is a medium-sized, thorny plant grows in the cold arid regions of Europe and Asia. Medicinal applications of these plants have been mentioned in the ancient Greek and Tibetan medicinal texts. Due to its wide medicinal applications, SBKT has also been named as a “wonder plant.” Different parts of the SBKT plants (berries, leaves, young shoots, roots, and bark) have been used in therapeutic purposes (Redei et al., 2018). In modern times, the SBKT plant and its parts have been studied for their therapeutic applications in cardiovascular, gastrointestinal, liver, and skin diseases (Zeb, 2004; Ganju et al., 2005; Gupta et al., 2005; Suchal et al., 2016; Hou et al., 2017). The oil content of the dried fruit pulp of SBKT is about 20–25%. The oil obtained from the pulp of the SBKT plant is composed of sterols, saturated and unsaturated fatty acids, tocopherols, tocotrienols, and carotenoids (Zeb, 2006; Bal et al., 2011; Bonesi et al., 2016; Zielinska and Nowak, 2017). Presence of high quantities of these biomolecules along with mineral elements like Ca, P, Fe, and K; vitamins like C, B1, B2, and K; and sugars like glucose and fructose, effectively presents the SBKT plant as a valuable source for human nutrition (Christaki, 2012). Omega-3 and omega-6 fatty acids have positive effects on neurological disorders, with observed anti-inflammatory and anti-oxidant activities (Kumar et al., 2011). Acute and sub-chronic toxicity studies in animals have also shown that SBKT is non-toxic up to 90 days of repeated dosing in rats (Zhao et al., 2017).

In the present study, we performed the chemical analysis of SBKT and tagged those findings with its observed biological efficacies. We also investigated the mechanism of action for the SBKT in its anti-inflammatory and anti-psoriasis-like activities in bacterial lipopolysaccharide (LPS)-stimulated human monocyte (THP-1) cells under *in vitro* conditions. Finally, we validated the efficacy of SBKT administered at a human equivalent dosage, in modifying Carrageenan-induced paw inflammation in Wistar rats, and in ameliorating 12-O-tetradecanoyl phorbol-13-acetate (TPA)-induced skin psoriasis in CD-1 mice.

## Materials and Methods

### Chemicals and Reagents

SBKT, isolated from the pulp, was obtained from Food and Herbal Park, Patanjali Ayurveda Limited, Haridwar, India. Culture media RPMI-1640, fetal bovine serum, and antibiotic/anti-mycotic mixture were obtained from Gibco. Cytokines interleukin 1-beta (IL-1β), IL-6, and tumor necrosis factor-α (TNF-α) ELISA kits were purchased from BD Biosciences. LPS, TPA, λ-Carrageenan, indomethacin (INDO), and dexamethasone (DEXA) were purchased from Sigma-Aldrich (St. Louis, MO, USA). Hematoxylin, potassium aluminium sulfate dodecahydrate, and mercury (II) oxide red were purchased from Merck India Pvt. Ltd, Mumbai, India. Eosin yellow and ferric chloride were purchased from Hi-Media Laboratories, Mumbai, India. All the other chemicals and reagents purchased for the study were of the highest commercial grade.

### Fatty Acid Profiling of SBKT

The saturated, polyunsaturated, and monounsaturated fat in SBKT were determined by gas chromatography–flame ionized detector (GC–FID) using AOAC Method 996.01 (AOAC, 2000). SBKT mixture was refluxed for 10 min after adding 10 ml methanolic NaOH solution. The mixture was again refluxed for an additional 5 min after the addition of 10 ml BF_3_ reagent. n-Heptane (10 ml) was added through the top of the condenser and refluxed for 1 min, and the reaction mixture was kept for 10 min at ambient temperature. The entire mixture was transferred to the centrifuge tube, mixed with 5 ml NaCl solution using a vortex at a low speed, and allowed to separate into multiple layers. One milliliter of the upper layer containing fatty acid methyl esters was transferred to GC vial and further used for the gas chromatography (AOAC, 2000).

For the GC–FID analysis of the SBKT fatty acid content, a 7890B gas chromatograph (Agilent Technologies) equipped with flame ionization detector and non-bonded 90% cyanopropyl and 10% phenyl siloxane capillary column was used. Measurements were obtained following the GC–FID operational parameters of injector temperature of 250°C, detector temperature of 275°C, H flow of 34 ml/min, air flow of ca. 300 ml/min, split ratio of 100:1, carrier gas of helium, linear velocity of 21 cm/s at 175°C, initial temperature of 120°C (hold 4 min), rate of 5°C/min, final temperature of 230°C, and final time of 5 min. Fatty acid methyl ester (FAME) mix was used in the identification and quantification of individual fatty acids. For GC–FID analysis, FAME was reconstituted with 10 ml of n-hexane (AOAC, 2000).

### Cell Culture for *In Vitro* Experiments

THP-1 cell line was obtained from the National Centre for Cell Science, Pune, India, and cultured in RPMI-1640 media, supplemented with 10% heat-inactivated fetal bovine serum in the presence of penicillin–streptomycin (100 U/ml), sodium pyruvate (1 mM), and L-glutamine (4 mM). The cells were grown at 37°C in a 5% CO_2_ in a sterile environment.

### Cell Viability Analysis

SBKT oil was prepared as an emulsion in incomplete culture media (RPMI-1640). THP-1 cells were plated in a 96-well plate at a concentration of 10,000 cells per well in a 96-well plate. The cells were pre-incubated overnight and exposed to the SBKT oil at concentrations of 0.0, 1.56, 3.12, 6.25, 12.5, 25, and 50 µl/ml for a period of 24 h. At the end of the exposure time, cells were washed with 100 µl PBS. One hundred microliters of 0.5 mg/ml 3-(4,5-dimethylthiazol-2-yl)-2,5-diphenyltetrazolium bromide was added to each well, and the plates were incubated for 3 h at 37°C. At the end of the exposure period, the dye was removed. One hundred microliters of dimethyl sulfoxide (DMSO) was added, and the plates were placed on a shaker for 10 min. Absorbance of each well was read using the PerkinElmer Envision microplate reader at 595-nm wavelength, and cell viability percentage was calculated.

### Reactive Nitrogen Species Measurement

THP-1 cells were seeded in 96-well culture plates at a density of 2 × 10^5^ cells/ml. Cells were treated with different concentrations of SBKT oil emulsion made in incomplete RPMI-1640 media and incubated for 1 h. Cells were stimulated with LPS (500 ng/ml) and incubated for an additional 24 h at 37ºC in CO_2_ incubator. The reactive nitrogen species (RNS) release in the culture media was determined using modified Griess reagent (Sigma), following the manufacturer’s protocol. Absorbance was recorded at 540 nm using Envision Microplate reader (PerkinElmer).

### Cytokines Level Measurement

THP-1 cells were seeded in 24-well culture plates at a density of 5 × 10^5^ cells/well. For the experiment, SBKT oil was prepared as an emulsion and mixed with the cell culture media at different concentrations: 1.25, 2.5, and 5 μl/ml. THP-1 cells were pre-incubated with the SBKT containing media for 1 h before addition of 1 µg/ml (final concentration) LPS. No LPS was added to the negative control cells. Cell culture supernatants were collected after 24 h, and different pro-inflammatory cytokines IL-1β, IL-6, and TNF-α were measured using ELISA kits (BD Biosciences) following the manufacturer’s protocol. Absorbance was recorded at 450 nm using the Envision microplate reader (PerkinElmer).

### Luciferase Reporter NF-κB Gene Assay

THP-1 cells were transiently transfected with luciferase reporter vector with NF-κB promoter sequence upstream of the luciferase gene. Transfection was performed following the manufacturer’s instruction in 96-well plates using Lipofectamine 3000 (Invitrogen, USA). Two days after transfection, the experiment was performed as described by Ishimoto et al. (2015) with the following modifications. Used media was replaced with media containing test compound and control. After 1 h, LPS was added at a concentration of 500 ng/ml, where required and incubated further for 12 h. D-Luciferin salt (PerkinElmer) at a final concentration of 150 μg/ml was added to the cells and incubated at 37°C, protected from light. Relative percentage changes in light emission intensity were measured from each well and calculated, and LPS alone was measured as 100% activity of the NF-κB reporter gene.

### Experimental Animals

CD-1 male mice (6–8 weeks) were procured from a Charles River Laboratory-licensed supplier, Hylasco Biotechnology Pvt. Ltd, Hyderabad, India. Male Wistar rats (8 to 10 weeks) were procured from Liveon Biolabs Pvt. Ltd, Bangalore, India. All the animals were placed under a controlled environment with a relative humidity of 60–70% and 12:12-h light and dark cycle in a registered animal house (1964/PO/RC/S/17/CPCSEA) of Patanjali Research Institute, India. The animals were fed a standard pellet diet (Golden Feed, India) and sterile-filtered water *ad libitum*. The study protocol was approved by the Institutional Animal Ethical Committee (IAEC) of Patanjali Research Institute vide approval numbers: PRIAS/LAF/IAEC-008 and PRIAS/LAF/IAEC-022. All the experiments were performed in accordance with relevant guidelines and regulations described by the ethical committees.

### Evaluation of *In Vivo* Anti-Inflammatory and Anti-Psoriasis-Like Efficacies

#### Carrageenan-Induced Rat Paw Edema Model

Carrageenan-induced paw edema test was performed according to the modified methods described earlier (Sakat et al., 2014). Wistar rats were divided into different groups of eight animals each based on basal paw volume (0 h), measured using Plethysmometer (Ugo Basile, Italy). Inflammation was induced by the subcutaneous injection of λ-Carrageenan (0.1 ml of 1% solution in normal saline) into the plantar side of the left hind paw. The paw was marked with ink at the level of the lateral malleolus, and the volume was measured up to the mark at 1, 2, 3, 4, and 5 h after carrageenan injection for all the animals. Further, animals were treated orally with SBKT [100 mg/kg p.o. + 40 μl/paw topical application (T.A.)] or INDO at 10 mg/kg (p.o.), 1 h before carrageenan challenge. Paw edema was calculated by subtracting the 0-h (basal) paw volume from the respective paw volumes at 1, 2, 3, 4, and 5 h. The anti-inflammatory activity (%) was calculated for each animal using the following formula: [Mean paw edema of control animals (ml) − paw edema of each test animals (ml)]/[Mean paw edema of control animals (ml)] × 100.

#### TPA-Induced Psoriasis-Like Lesion Mouse Model

Anti-psoriatic-like effects of SBKT were examined on the TPA-induced skin inflammation model as described previously with a slight modification (Goto et al., 2010). Briefly, 20 µl of TPA solution (2.5 μg/ear of TPA in acetone) was applied topically on the right ear of CD-1 mice on days 0, 2, 4, 6, 8, and 10. The left ear was served as the vehicle control and treated with 20 µl of acetone on the same days. Ear thickness was measured every day using a digital Vernier caliper (Mitutoyo, Tokyo, Japan). An increase in ear thickness was determined by subtracting the ear thickness of day 0 (before TPA or acetone application) from the respective time point thickness. Animals were treated with a vehicle or SBKT (at 100 mg/kg p.o. + 20 μl T.A and 200 mg/kg p.o. + 20 μl T.A.) or DEXA (0.2 mg/ear T.A.) throughout the study. The anti-psoriasis activity (%) was calculated for each animal on day 10 (D10), using the following formula: [Mean ear edema of TPA control mice − ear edema of each mouse of test or DEXA-treated mouse]/[Mean ear edema of TPA control mice] × 100.

#### Histopathological Analysis

CD-1 mice were humanely euthanized on day 10 after 6 h of the last drug treatment. Ear biopsy samples were weighed and fixed in 10% (v/v) neutral-buffered formalin, embedded in paraffin, and sectioned at 3–5 μm. The sections were then stained with hematoxylin and eosin. By using a bright-field microscope, low-magnification and high-magnification histology images of the ear biopsy samples were obtained at 100× and 400×, respectively. The thickness of the epidermis (from the basal layer to the stratum corneum) was measured by MagVision image analysis software using the Magcam DC5 microscopic camera and calibration by a stage micrometer. The severity of the observed lesions was recorded as NAD = no abnormality detected, 1 = minimal (<1%), 2 = mild (1–25%), 3 = moderate (26–50%), 4 = moderately severe/marked (51–75%), and 5 = severe (76–100%). Distribution of the lesions was recorded as focal, multifocal, and diffused. The different parameters like the extent of the lesion, severity of hyperkeratosis, number and size of pustules, epidermal hyperplasia (measured in the interfollicular epidermis), the severity of inflammation in the dermis and soft tissue, and any other lesion(s) were considered for histopathological examination and scoring.

### Statistical Analysis

The data are expressed as mean ± SEM for each group. Statistical analysis was done using GraphPad Prism version 7.03 software. Two-way ANOVA followed by Newman–Keuls multiple comparison test was used to calculate the statistical difference in absolute paw volume, paw edema, and ear edema. A one-way ANOVA followed by Dunnett’s multiple comparison post hoc test was used to calculate the statistical difference in cytokine(s) analysis, ear biopsy weights, epidermal thickness, and lesion scores. The values of p < 0.05 were considered statistically significant.

## Results

### Chemical Profiling of SBKT Components Using GC–FID

**Figure 1:**
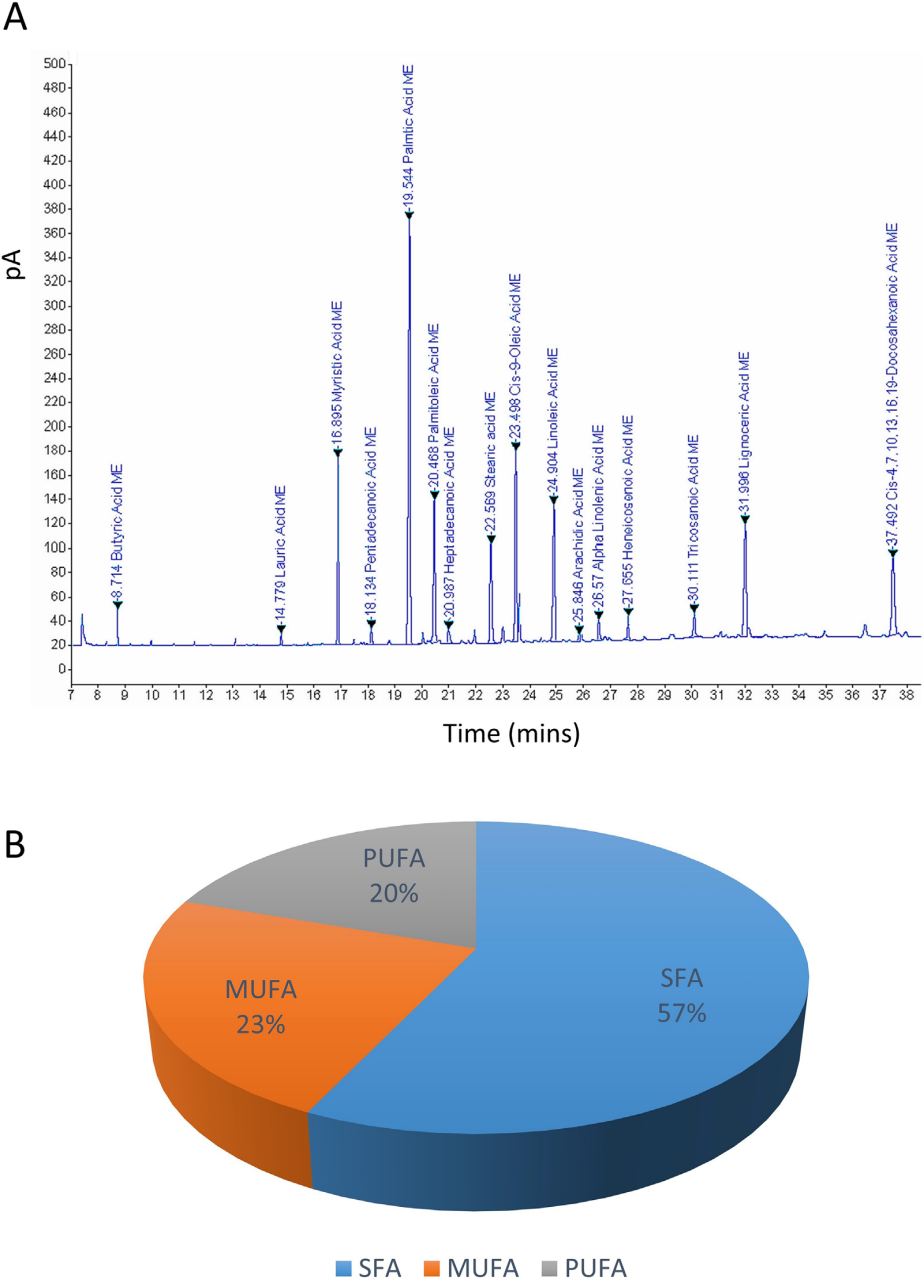
Gas chromatography–flame ionized detector (GC–FID) chromatogram of sea buckthorn oil (SBKT). **(A)** The fatty acid composition of the SBKT was determined using the GC–FID methodology. Individual fatty acids were identified and quantified using fatty acid methyl ester methodology. Chromatography analysis identified 16 major fatty acids. **(B)** Pie-diagram represents the percentage of three categories of fatty acids identified in the SBKT—saturated fatty acids (SFA), monounsaturated fatty acids (MUFA), and polyunsaturated fatty acids (PUFA) (also see **Table 1**).

GC–FID technique-based chemical analysis of the SBKT showed the presence of 16 major fatty acid peaks and several minor peaks (**Figure 1A**). Saturated fatty acid content represented the highest quantity of fatty acids (57.06%) present in the SBKT, followed by monounsaturated (23.31%) and polyunsaturated (19.64%) fatty acids (**Table 1** and **Figure 1B**). FAME-based GC–FID analysis of the SBKT for the identification and quantification of individual fatty acids showed the presence of palmitic acids (26.30%), cis-9 oleic acid (13.66%), linoleic acid (9.31%), lignoceric acid (9.16%), myristic acid (8.40%), palmitoleic acid (8.10%), stearic acid (7.45%), tricosanoic acid (1.97%), henicosadienoic acid (1.55%), alpha-linolenic acid 1.53%), heptadecanoic acid (1.31%), butyric acids (1.12%), pentadecanoic acid (0.81%), and arachidic acid (0.54%) (**Table 1**). Several other fatty acid components were also detected in the SBKT, but their quantities were relatively low (<0.10%) (**Table 1**).

**Table 1:** Gas chromatography–flame ionized detector analysis of sea buckthorn oil (SBKT) fatty acid contents.

		
Palmitic acid ME (C_16_H_32_O_2_; mol wt. 256.43; SFA)	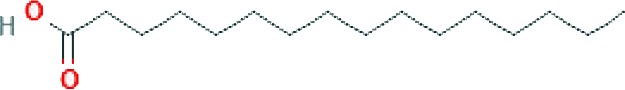	26.30
Lignoceric acid ME (C_24_H_48_O_2_; mol wt. 368.63; SFA)	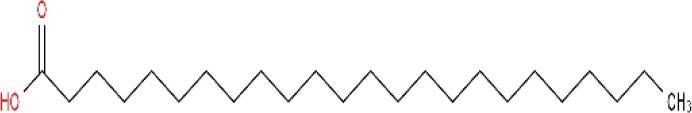	9.16
Myristic acid ME (C_14_H_28_O_2_; mol wt 228.37; SFA)	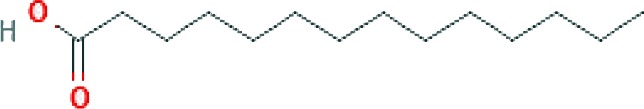	8.40
Stearic acid ME (C_18_H_36_O_2_; mol wt. 284.48; SFA)	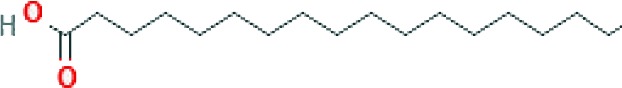	7.45
Tricosanoic acid ME (C_23_H_46_O_2_; mol wt. 354.61; SFA)	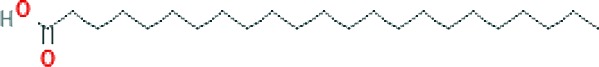	1.97
Butyric acid ME (C_4_H_8_O_2_; mol wt. 88.11; SFA)	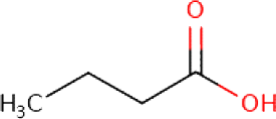	1.12
Heptadecanoic acid ME (C_17_H_34_O_2_; mol wt 270.45; SFA)	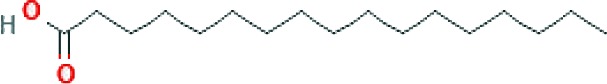	1.31
Pentadecanoic acid ME (C_15_H_30_O_2_; mol wt. 242.39; SFA)	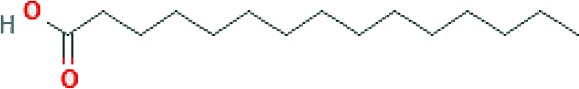	0.81
Arachidic acid ME (C_20_H_40_O_2_; mol wt. 312.53; SFA)	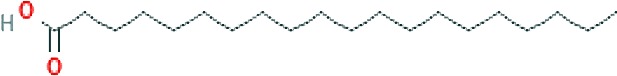	0.54
Cis-9 Oleic acid ME (C_18_H_34_O_2_; mol wt. 282.47; MUFA)	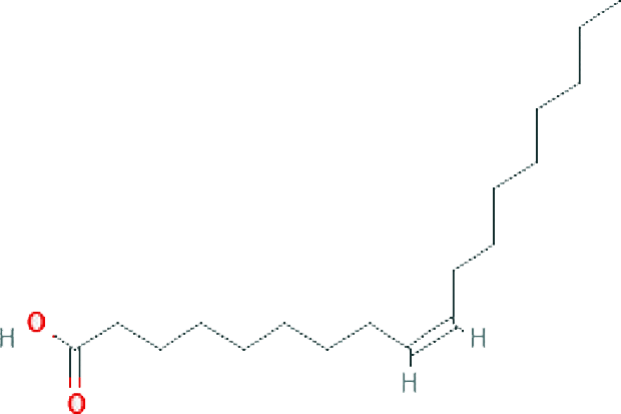	13.66
Palmitoleic acid ME (C_16_H_30_O_2_; mol wt. 254.41; MUFA)	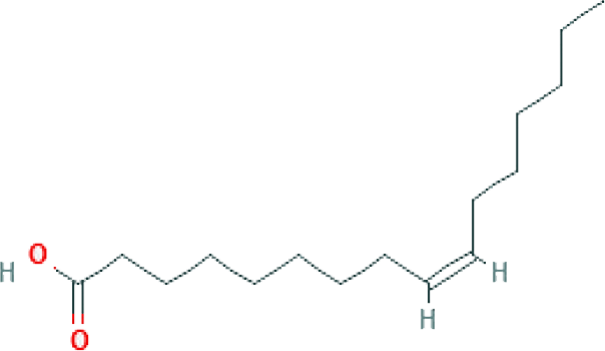	8.10
Henicosadienoic acid ME (C_21_H_38_O_2_; mol wt. 322.53; MUFA)	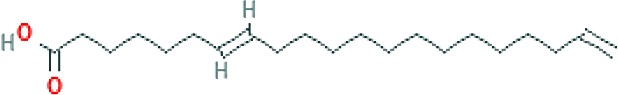	1.55
Linoleic acid ME (C_18_H_32_O_2_; mol wt. 280.44; PUFA)	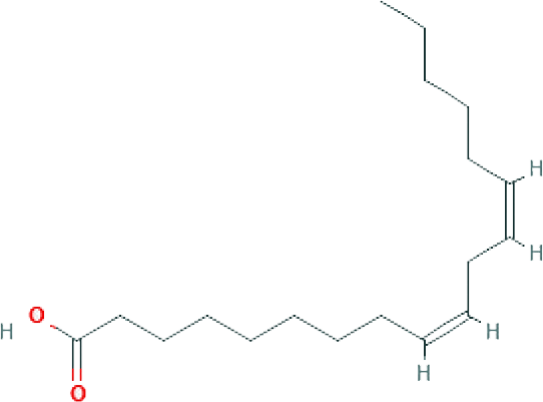	9.31
Docosahexaenoic acid ME (C_22_H_32_O_2_; mol wt 328.48; PUFA)	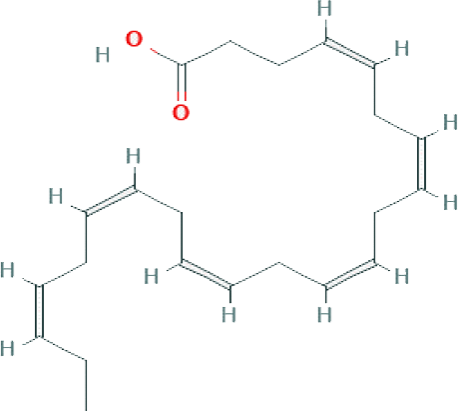	8.80
Alpha-linolenic acid ME (C_18_H_30_O_2_; mol wt. 278.43; PUFA)	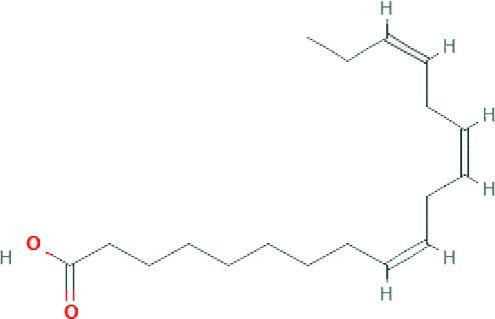	1.53

Quantified fatty acid methyl esters (ME) have been listed in the descending order of contents (%) for saturated fatty acids (SFA), monounsaturated fatty acids (MUFA), and polyunsaturated fatty acids (PUFA), in SBKT (also see **Figure 1**). The following fatty acid MEs were found to be <0.10% quantitatively: caproic acid ME, cis-10-penta decanoic acid ME, cis-10-heptadecanoic acid ME, trans-9 elaidic acid ME, linolelaidic acid ME, myristoleic acid ME, cis-11-eicosenoic acid ME, caprylic acid ME, cis-11,14 eicosadienoic acid ME, behenic acid ME, cis 8,11,14 eicosatrienoic acid ME, erucic acid ME, cis 11,14,17 eicosatrienoic acid, capric acid ME, lauric acid ME, tridecanoic acid ME, EPA ME, gamma-linolenic acid ME, arachidonic acid ME, cis 13,16 docosadienoic acid ME, and nervonic acid ME.

### *In Vitro* Anti-Inflammatory Activity of SBKT

**Figure 2:**
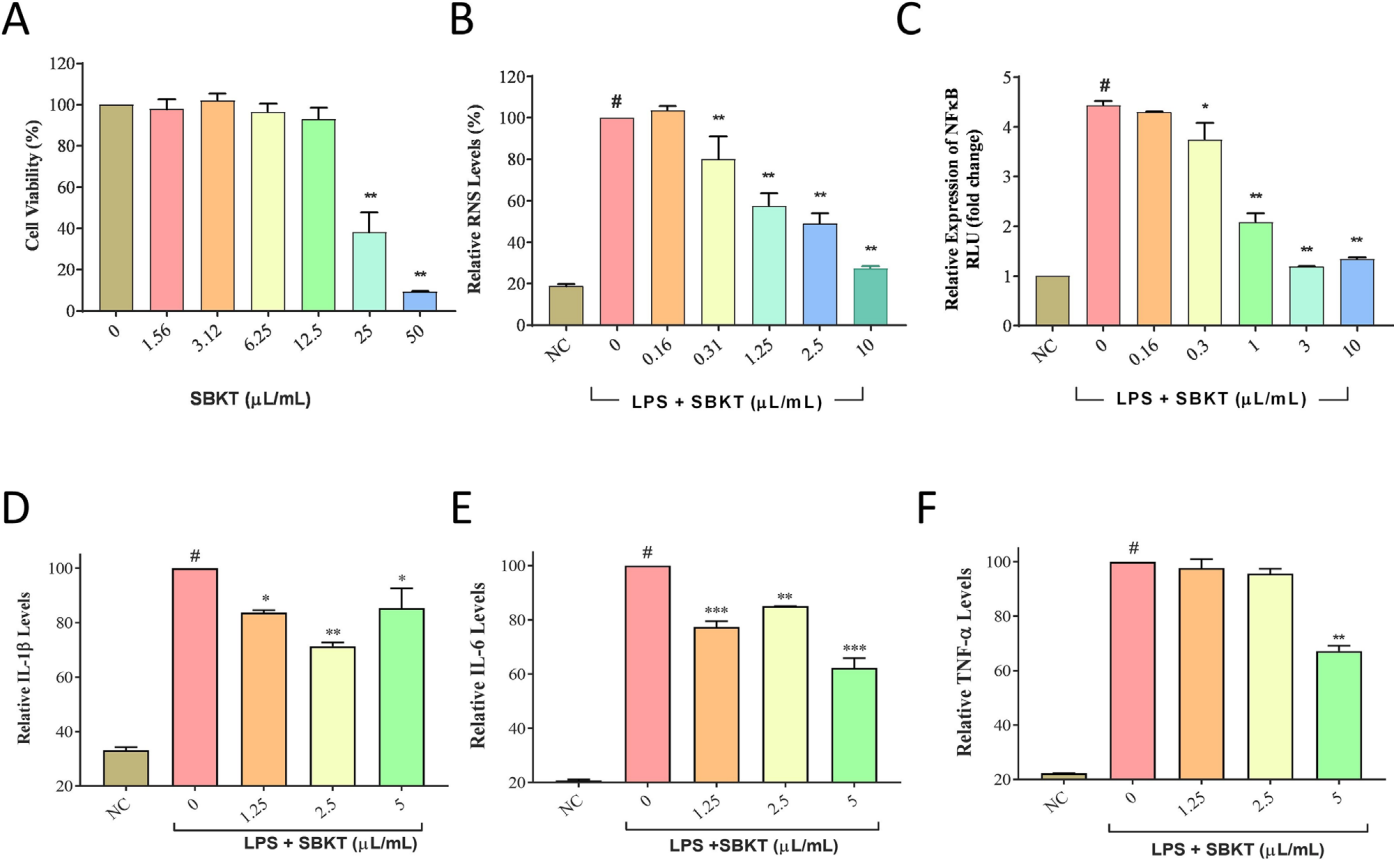
*In vitro* anti-inflammatory potentials of the SBKT. **(A)** Treatment of THP-1 cells with SBKT induced loss of cell viability at concentrations ≥25 μl/ml. Treatment of the lipopolysaccharide (LPS)-stimulated THP-1 cells with the non-cytotoxic doses of SBKT showed amelioration in the production of **(B)** reactive nitrogen species (RNS) and **(C)** NF-κB. Downstream reduction in the release of pro-inflammatory cytokines: **(D)** interleukin-1ß (IL-1ß), **(E)** interleukin-6 (IL-6), and **(F)** tumor necrosis factor-α (TNF-α) was observed in the LPS-stimulated THP-1 cells treated with different concentrations of SBKT. These experiments were performed thrice in triplicates. One-way ANOVA followed by Dunnett’s post hoc test was performed to calculate statistical significance. ^#^p < 0.001 (LPS-stimulated *versus *normal control cells); *p < 0.05, **p < 0.01, ***p < 0.001 (LPS only stimulated cells *versus* LPS + SBKT-treated cells).

Traditional use of SBKT in reducing gastric ulcers as an anti-inflammatory mediator has been attributed to the modulation of pro-inflammatory mediators (Xing et al., 2002). SBKT was found to induce significant (p < 0.01) loss of cell viability in the normal THP-1 cells at concentrations ≥25 μl/ml (**Figure 2A**). Therefore, 10 μl/ml was taken as the maximum test dose for subsquent *in vitro* assessments. Analysis of the RNS production in the LPS-stimulated THP-1 cells showed a significant (p < 0.001) increase as compared to the normal cells. SBKT treatment of the stimulated THP-1 cells led to a reduction in the cellular RNS levels (p < 0.01) in a concentration-dependent manner (**Figure 2B**). Treatment of the THP-1 cells with LPS also stimulated the expression of inflammatory NF-κB protein (**Figure 2C**). This stirred-up increase in the NF-κB protein expression was ameliorated by SBKT treatment. Increase in the NF-κB protein expression was also associated with a significant increase in the release of the pro-inflammatory cytokines: IL-1β (p < 0.001), IL-6 (p < 0.001), and TNF-α (p < 0.001) (**Figures 2D, E, F**). Treatment of the THP-1 cells with SBKT emulsion significantly reduced the LPS-stimulated release levels of IL-1β [5 μl/ml (p < 0.05), 2.5 μl/ml (p < 0.01), and 1.25 μl/ml (p < 0.05)] and IL-6 [5 μl/ml (p < 0.001), 2.5 μl/ml (p < 0.01), and 1.25 μl/ml (p < 0.001)] from the THP-1 cells (**Figures 2D, E**). A reducing trend in TNF-α cytokine release in the THP-1 cells treated with SBKT and LPS was observed at 24 h, with a significant reduction occurring at the highest test concentration of the oil (5 μl/ml; p < 0.01) (**Figure 2F**).

### *In Vivo* Anti-Inflammatory Effects of SBKT

**Figure 3:**
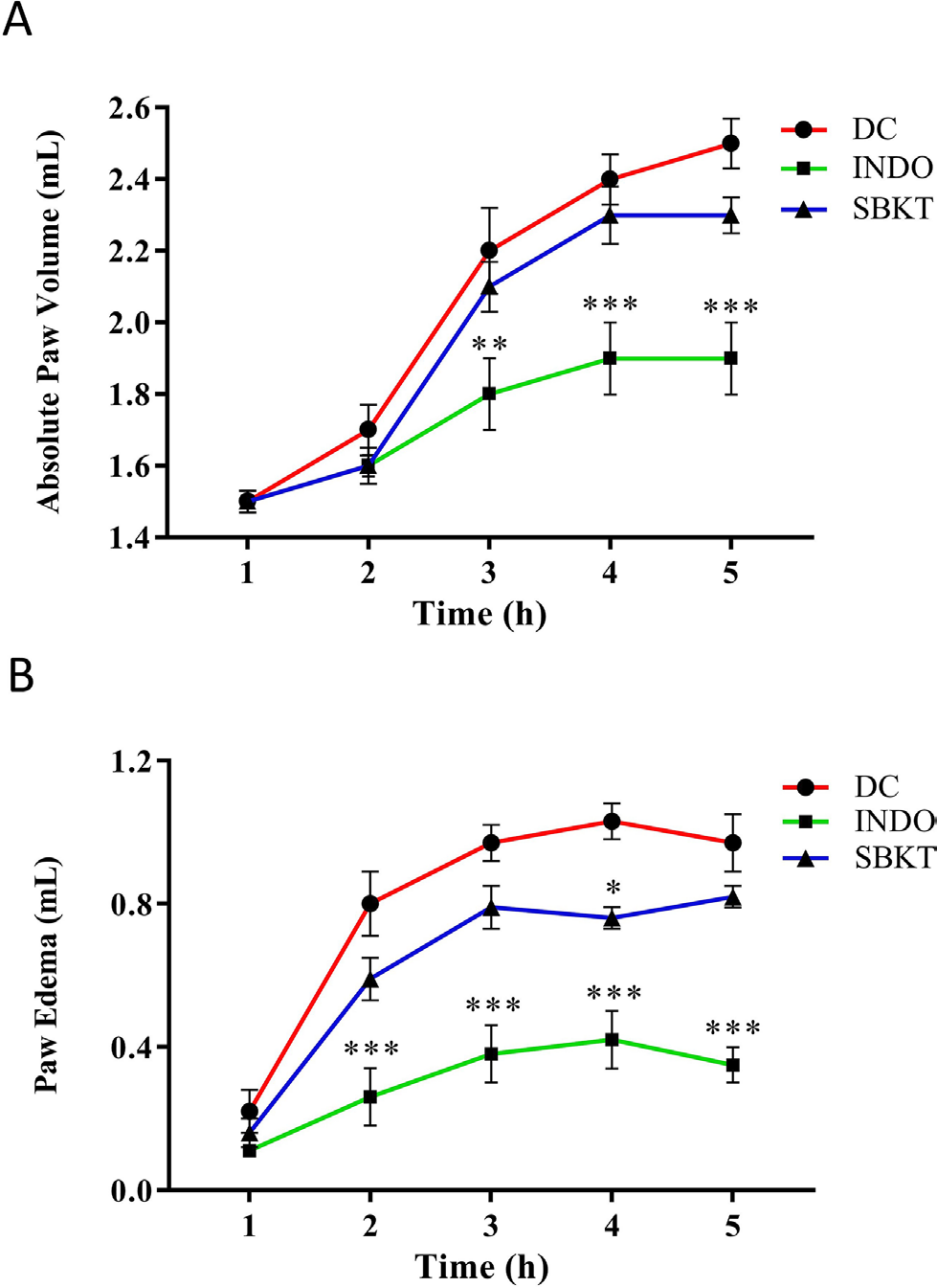
Effect of SBKT on λ-Carrageenan-induced paw rat edema model. Co-treatment of carrageenan-stimulated Wistar rats with SBKT [100 mg/kg; p.o. and 40 μl topical application (T.A.)] or with INDO (10 mg/kg; p.o.) led to a considerable reduction in their **(A)** absolute paw volume and **(B)** paw edema. Statistical analysis of the treatments was performed using two-way ANOVA followed by Newman–Keuls multiple comparison test (n = 7 animals). *p < 0.05, **p < 0.01, ***p < 0.001 (DC *versus* SBKT; DC *versus* INDO-treated animals). DC, disease control, SBKT, sea buckthorn oil; INDO, indomethacin.

Subplantar injection of λ-Carrageenan (0.1 ml of 1% solution in normal saline) in the Wistar rats induced a time-dependent increase in both the absolute paw volume and paw edema (**Figures 3A, B**). Post-treatment of the Carrageenan-stimulated Wistar rats with 10 mg/kg of standard anti-inflammatory drug INDO exhibited a significant reduction of absolute paw volume (p < 0.001) and paw edema (p < 0.001) (**Figures 3A, B**). Oral (100 mg/kg: calculated from a human equivalent dose of 2000 mg/day, for rats) and topical (40 µl/paw) treatment of the Carrageenan-stimulated rats with SBKT induced an observable decrease in both the paw absolute volume and paw edema (statistically significant at 4 h; p < 0.05), compared to the disease control animals (**Figures 3A, B**).

### *In Vivo* Anti-Psoriatic Activity of SBKT

**Figure 4:**
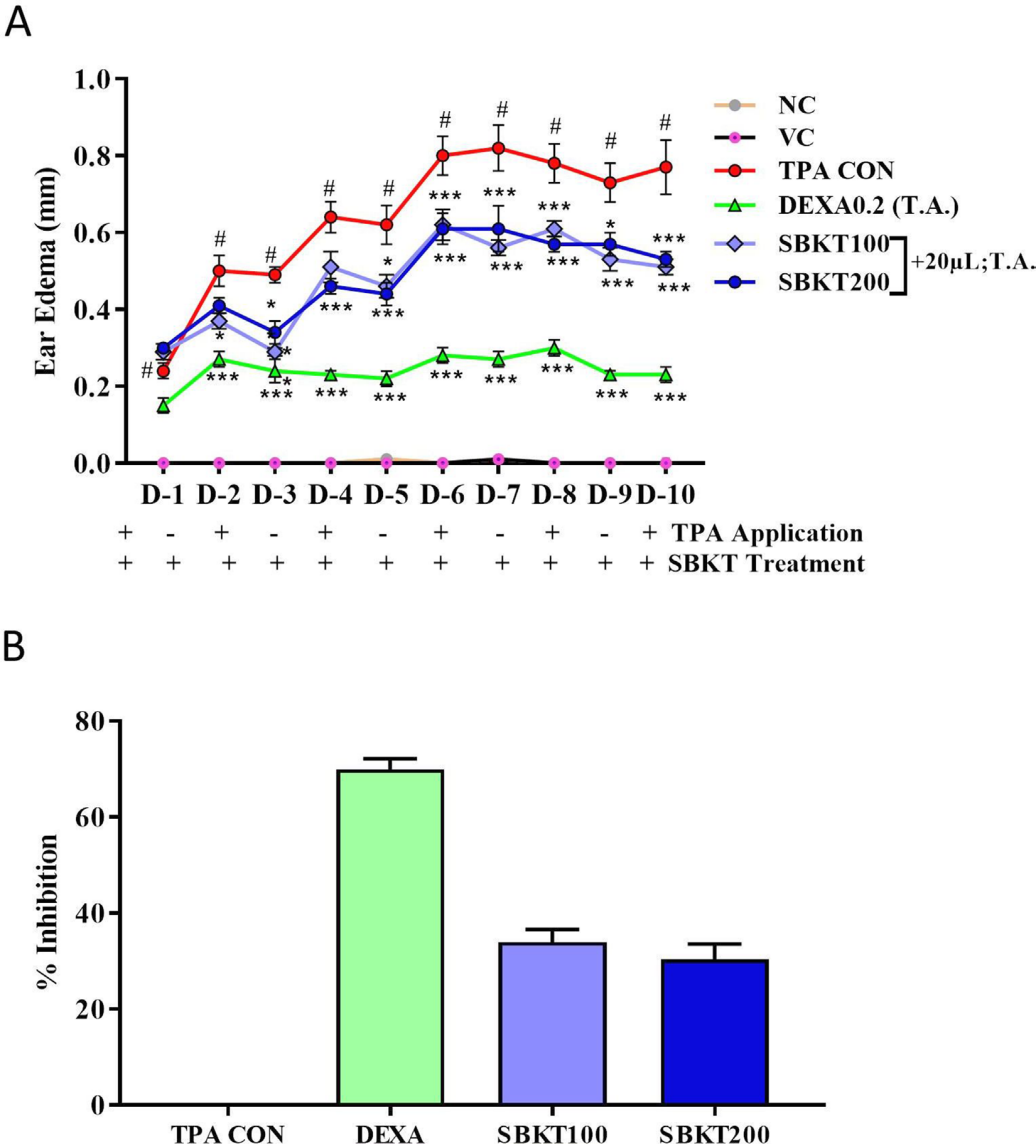
Effect of SBKT on TPA-induced ear edema in mice. **(A)** Co-treatment of the TPA-stimulated psoriatic ear (TPA CON) with SBKT (100 and 200 mg/kg; p.o. and 20 μl T.A.) or with DEXA (0.2 mg/ear; T.A.) significantly reduced ear edema. **(B)** Represents percent ear edema inhibition (activity) calculated in comparison to TPA CON animals at D10. This panel represents % inhibition (activity) of SBKT 100, SBKT 200, and DEXA individually. Statistical analysis was performed using two-way ANOVA followed by Newman–Keuls multiple comparison test (n = 8 animals). ^#^p < 0.001 (NC *versus* TPA CON); NS p > 0.05 (NC versus VC) *p < 0.05, **p < 0.01, ***p < 0.001 (TPA CON *versus* SBKT; TPA CON *versus* DEXA). NC, normal control; VC, vehicle control; TPA CON, 12-O-tetradecanoyl phorbol-13-acetate; DEXA, dexamethasone; SBKT, sea buckthorn oil.

TPA formulated in acetone was applied on the CD-1 mouse ear (2.5 µg/ear) for induction of psoriasis-like disease. Topical application of TPA significantly induced ear edema in the control (TPA CON) animals (p < 0.001) (**Figure 4A**). Topical treatment of the psoriatic ear with the standard anti-inflammatory drug DEXA (0.2 mg/ear) significantly reduced the ear edema from day 2 onward (p < 0.001) (**Figure 4A**). Similarly, concurrent oral (100 and 200 mg/kg) and topical (20 µl) treatment of the SBKT in the psoriatic animals significantly reduced ear edema from day 2 onwards. In these test parameters, no significant variation in the responses was observed in the animals treated with 100 and 200 mg/kg-dose of SBKT. For mice, 200 mg/kg is the calculated human equivalent dose (2000 mg/day), as per body weights and surface area conversions. The percent inhibition (at D-10) in the ear edema of DEXA and SBKT 100 and 200 mg/kg treated mice was found to be 70.05 ± 6.25%, 34.05 ± 7.65%, and 30.45 ± 8.90%, respectively, in comparison to TPA CON mice (**Figure 4B**).

### Effect of SBKT on Psoriatic Ear Biopsy Weight and Epidermal Thickness

**Figure 5:**
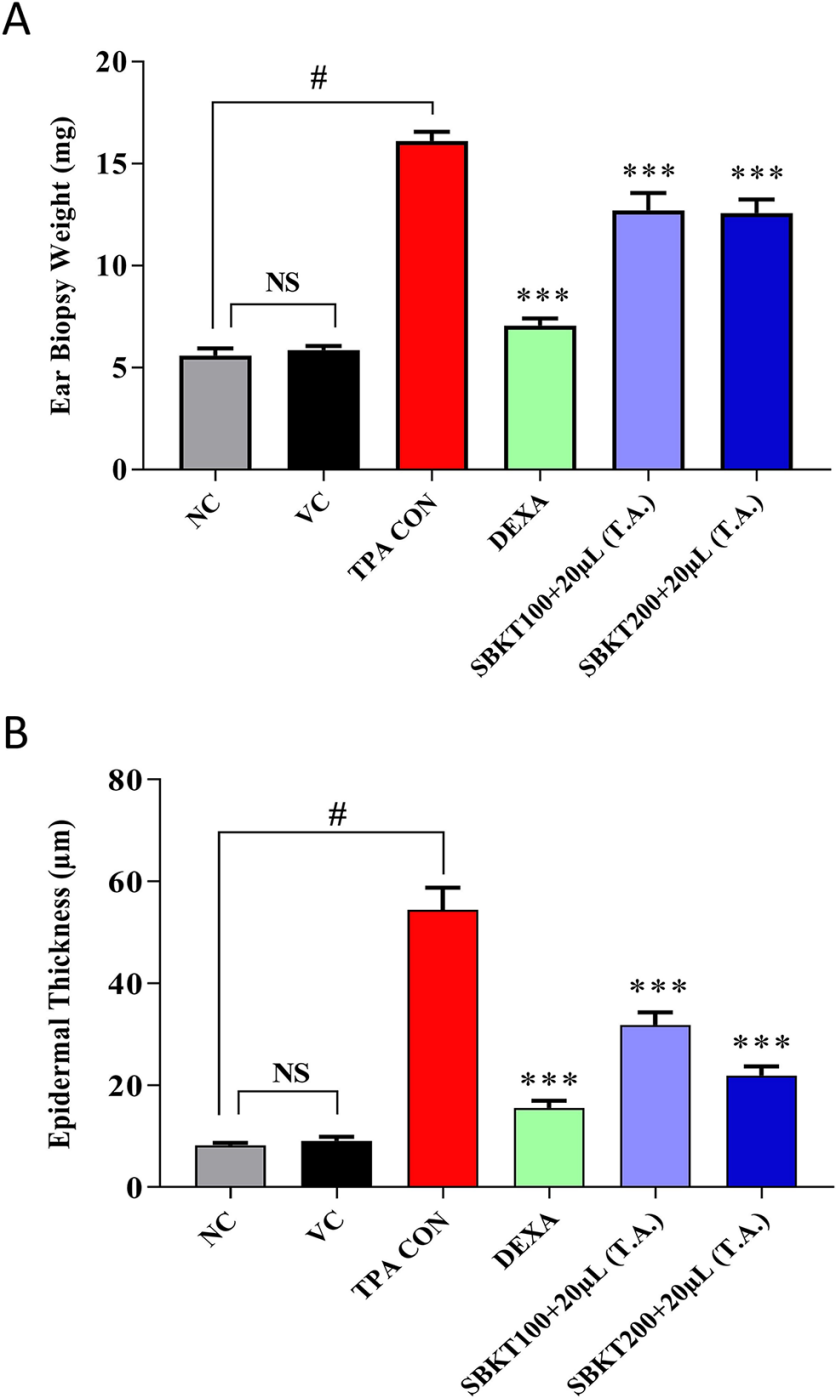
Effect of SBKT on TPA-induced ear biopsy weight in mice. Co-treatment of the psoriatic ear with SBKT (100 and 200 mg/kg; p.o. and 20 μl T.A.) or with DEXA (0.2 mg/ear; T.A.) significantly reduced the **(A)** ear biopsy weight and **(B)** ear epidermal thickness. Statistical analysis was performed using one-way ANOVA method followed by Dunnett’s multiple comparison *t* test (n = 8 animals). ^#^p < 0.001 (NC *versus* TPA CON); NS p>0.05 (NC versus VC); ***p < 0.001 (TPA CON *versus* SBKT; TPA CON *versus* DEXA). NC, normal control; VC, vehicle control; TPA CON, 12-O-tetradecanoyl phorbol-13-acetate; DEXA, dexamethasone; SBKT, sea buckthorn oil.

Increased ear biopsy weight was detected in the TPA CON CD-1 mice after 10 days’ treatment showing inflammatory effects (p < 0.001) (**Figures 5A, B**). Treatment of the psoriatic animals with DEXA (0.2 mg/ear) significantly reduced the elevated ear biopsy weight (p < 0.001) (**Figure 5A**). Oral (100 and 200 mg/kg) and topical (20 μl) treatment of the SBKT also significantly reduced the inflammation-induced biopsy weight (p < 0.001) compared to the TPA CON animal (**Figure 5A**).

Similarly, histopathological evaluation of psoriatic ear punch biopsy (TPA CON) showed a significant (p < 0.001) increase in epidermal thickness (54.42 ± 12.20 μm) as compared to the normal control (NC) animals (8.26 ± 1.07 μm) (**Figure 5B**). Treatment of the TPA-induced psoriatic ear with the topical application of DEXA significantly (p < 0.001) reduced the epidermal layer thickness (15.53 ± 4.10 μm). Similarly, concurrent oral (100 and 200 mg/kg) and topical (20 μl) treatment of the SBKT also significantly (p < 0.001) reduced the ear epidermal thickness (31.80 ± 6.90 μm and 21.91 ± 5.07 μm, respectively), indicating the anti-inflammatory and anti-psoriatic efficacies of SBKT (**Figure 5B**).

### Effect of SBKT on Inflammatory Lesion Scores

**Figure 6:**
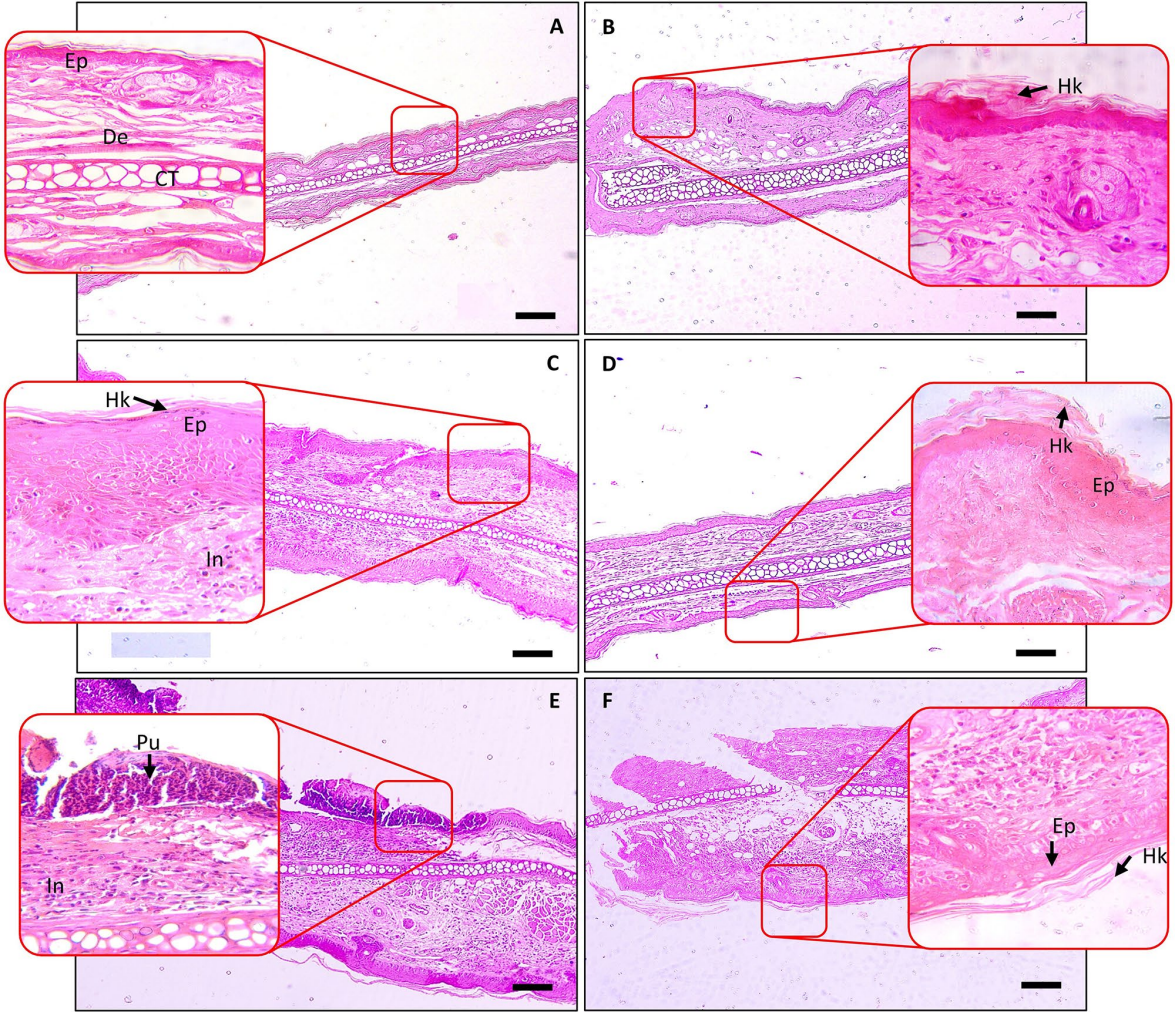
Histopathological analysis of SBKT treatment on TPA-induced ear psoriasis in mice. Histopathological analysis of mice ear tissue was performed following fixation and hematoxylin and eosin staining. Low-magnification images were obtained at 100×, and the higher-magnification image was obtained at 400×. **(A)** Normal control: represents normal epidermis (Ep), dermis (De), sebaceous gland (Sg), cartilage (CT). **(B)** Vehicle control (acetone) treated ear: represents normal epidermis (Ep), dermis (De), sebaceous gland (Sg), cartilage (CT). **(C)** TPA-CON: represents hyperkeratosis (Hk) and hyperplastic epidermis (Ep), presence of inflammatory cells (In) in the dermis region. **(D)** TPA and DEXA (0.2 mg/ear) treated ear: reduced hyperplastic epidermis (Ep), absence of inflammatory cells in the dermis region. **(E)** TPA and SBKT (100 mg/kg; p.o. and 20 μl; T.A.) treated ear: reduced hyperkeratosis (Hk) and hyperplastic epidermis (Ep), reduced presence of inflammatory cells (In) in the dermis region. **(F)** TPA and SBKT (200 mg/kg; p.o. and 20 μl; T.A.) treated ear: reduced hyperkeratosis (Hk) and hyperplastic epidermis (Ep). The scale represents 100 µm (n = 8 animals).

Histopathological analysis of the TPA-stimulated mice ear showed a significant increase in inflammatory lesions score such as epidermal hyperkeratosis and infiltration of inflammatory cells in the dermal region (**Figures 6A, C**). No such changes were observed in the tissue of the mouse ears treated with vehicle control (**Figure 6B**). Treatment of the TPA-stimulated ear with DEXA reduced the influx of inflammatory cells but continued to show the signs of persisting hyperkeratosis lesions and hyperplasticity in the epidermis (**Figure 6D**). Concurrent oral (100 and 200 mg/kg) and topical (20 μl) application of the SBKT on the psoriatic mice ear also reduced signs of hyperkeratosis and hyperplasticity in the skin epidermis but sustained the presence of inflammatory cells in the dermal region (**Figures 6E, F**).

**Figure 7:**
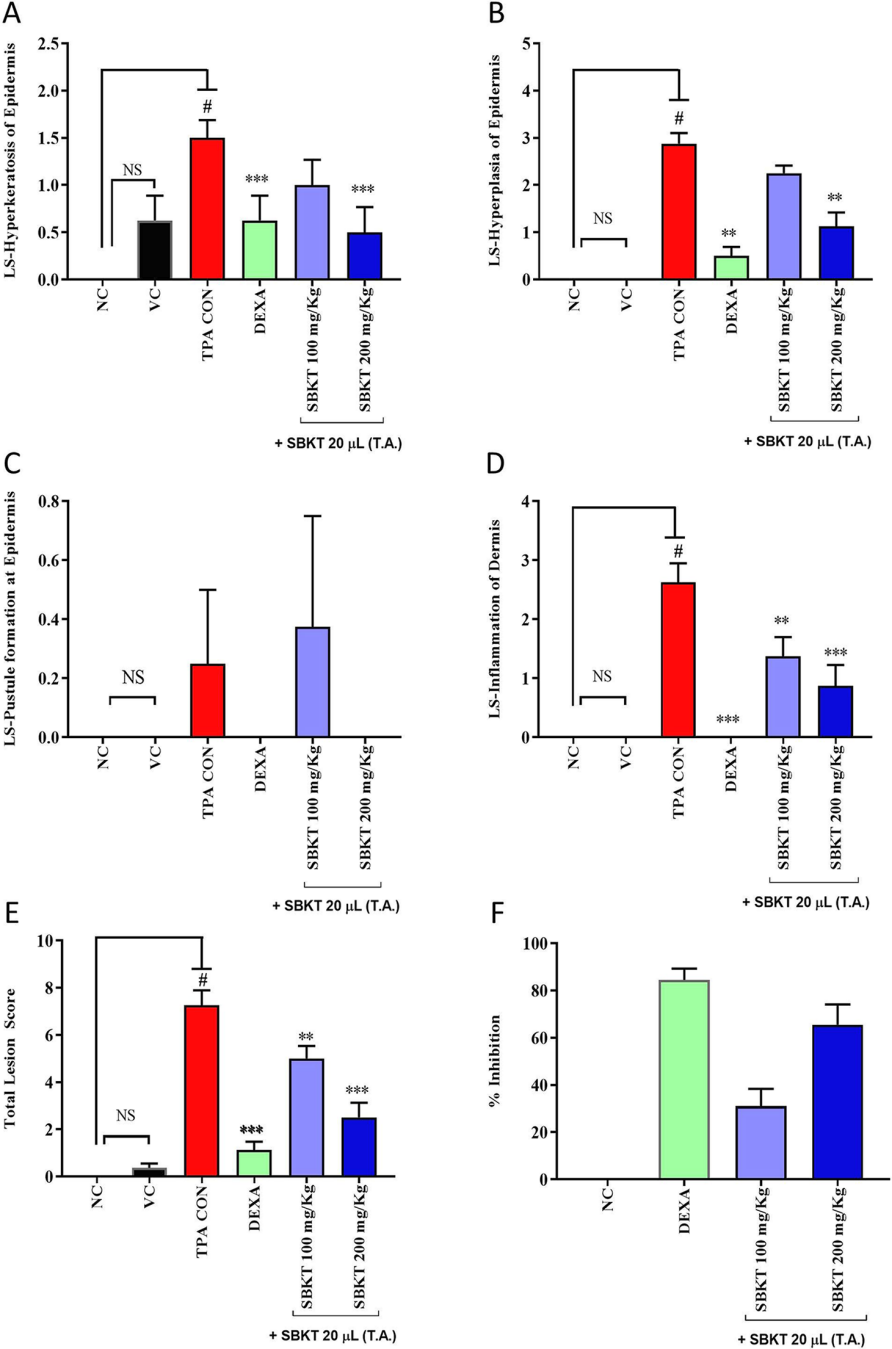
Effect of SBKT on TPA-induced inflammatory lesions in mice ear. Mice co-treated with TPA and DEXA or SBKT showed a reduction in histopathological lesions viz. **(A)** Hyperkeratosis of the epidermis. The data clearly demonstrated the considerable decrease in hyperkeratosis score by SBKT at 100 and 200 mg/kg (p < 0.001) in comparison to TPA CON. **(B)** Hyperplasia of the epidermis. Results showed a decrease in hyperplasia score by SBKT at 100 and 200 mg/kg (p < 0.001). **(C)** Pustule formation at the epidermis. Only SBKT at 200 mg/kg (p < 0.001) was found to reduce pustule formation in comparison to TPA CON. **(D)** Inflammation of the dermis. A significant decrease in dermal inflammation score was observed following treatment with SBKT at 100 (p < 0.01) and 200 mg/kg (p < 0.001). **(E)** Results showed a significant decrease in total lesion score at 100 (p < 0.01) and 200 mg/kg (p < 0.001). Represents percentage inhibition of total lesion score. % Inhibition data have not been statistically compared between SBKT 100 and SBKT 200 with DEXA. This panel represents % inhibition (activity) of SBKT 100, SBKT 200, and DEXA individually. **(F)** Percentage inhibition of total lesion score. High activity in inhibiting inflammatory lesions was observed in the SBKT at 200 mg/kg followed by 100 mg/kg in comparison to TPA CON. Statistical analysis was performed using one-way ANOVA followed by Dunnett’s multiple comparisons *t* test (n = 8 animals). ^#^p < 0.001 (NC *versus* TPA CON), NS p > 0.05 (NC *versus* VC), **p < 0.01, ***p < 0.001 (TPA CON *versus* SBKT; TPA CON *versus* DEXA). NC, normal control; VC, vehicle control; TPA CON, TPA control; SBKT, sea buckthorn oil; DEXA, dexamethasone.

Individual scoring through histopathological analysis further confirmed the efficacy of SBKT. Results suggested an elevation in the lesion score of hyperkeratosis, epidermal hyperplasia, pustule formation, and inflammatory cell infiltration in the epidermal and dermal regions of the TPA CON animals (**Figures 7A–D**). Treatment of the psoriatic ear with oral and topical (20 μl) application of the SBKT exhibited a significant reduction in the lesion scores [hyperkeratosis: SBKT 200 mg/kg (p < 0.05); hyperplasia: SBKT 200 mg/kg (p < 0.001); pustule formation and epidermal inflammation: SBKT 100 mg/kg (p < 0.01) and SBKT 200 mg/kg (p < 0.001); inflammatory cells infiltration] (**Figures 7A–D**). DEXA topical treatment also reduced the observed individual lesion scores and infiltration of inflammatory cells (**Figures 7A–D**), as expected. Total lesion score analysis and % inhibition (activity) calculation showed a significant decrease in the overall inflammation in the SBKT (p < 0.01 at 100 mg/kg; p < 0.001 at 200 mg/kg) and DEXA (p < 0.001) treated TPA-stimulated mice (**Figures 7E, F**).

## Discussion

Psoriasis a common skin systemic inflammatory disease leading to the development of dermal changes such as itching, burning, and soreness (Lowes et al., 2007; Wagner et al., 2010). During the onset of disease, the affected keratinocytes and pro-inflammatory immune cell cross-talk to release soluble pro-inflammatory mediators such as IL-1β, TNF-α, IL-6, and IL-8. So far, there is no permanent cure for psoriasis, and the disease can undergo cyclic evolution with periods of flaring for weeks or months, and then becoming dormant for a significant amount of time.

SBKT has been used as a good source of nutrition for centuries (see Krejcarová et al., 2015, for the detailed review), with defined medicinal uses like cardioprotective, antidiabetic, hepatoprotective, and anti-carcinogenic activities. In the present study, we investigated the SBKT extracted from the fruit pulp for its anti-inflammatory and anti-psoriatic roles. Initial chemical analysis of the fatty acid content of the SBKT using GC–FID revealed the presence of high quantities of saturated, monounsaturated, and polyunsaturated fatty acids along with smaller factions of several other fatty acids. Presence of most of these fatty acids in the SBKT has been reported earlier by Zielinska and Nowak and correlates well with our findings (Zielinska and Nowak, 2017). Fatty acids such as palmitic acid, oleic acid, palmitoleic acid, stearic acid, and the linoleic acid identified in the SBKT act as major constituents of the human epidermis (Kim et al., 2010). Palmitoleic acid also promotes wound healing and diminishes inflammation through modulation of pro-inflammatory cytokines (Bal et al., 2011; Kumar et al., 2011; Shi et al., 2017; Souza et al., 2017). Omega-3 (α-linolenic acid) and omega-6 (linoleic acids) fatty acids present in the SBKT have been identified as essential components of the body and help in the translocation of the fat-soluble vitamins (A, D, E, and K) and wound healing (Lee et al., 2006; Cupara et al., 2011; Ito et al., 2014; Calder, 2017). Omega-3 (α-linolenic acid), omega-6 (linoleic acid), and omega-9 (oleic acid) fatty acids also help in forming a protective barrier against trans-epidermal water loss (Zielinska and Nowak, 2017). Therefore, the presence of these saturated, monounsaturated, and polyunsaturated fatty acid components in the SBKT along with other bioactive compounds helps in forming a protective barrier for the skin and helps in the skin wound healing and repair.

Inflammation plays a major role in the development of psoriasis. Initiation of psoriasis is associated with the infiltration of the pro-inflammatory cells such as, monocytes, neutrophils, and T cells (Ogawa et al., 2018). These cells boost the development of inflammation and oxidative stress through the release of pro-inflammatory cytokines and reactive oxygen and nitrogen species. Our initial *in vitro* screening for the anti-inflammatory behavior of SBKT was done using LPS-stimulated THP-1 cells. Treatment of the THP-1 cells with SBKT showed that it is capable of inducing cytotoxicity at a dose of 25 μl/ml. Applying a non-cytotoxic dose, SBKT was found capable of ameliorating LPS-induced inflammation in the THP-1 cells through the reduction of pro-inflammatory RNS levels and NF-κB protein expression. Both the RNS and NF-κB have been reported as critical components involved in the induction of psoriasis (Bruch-Gerharz et al., 1998; Goldminz et al., 2013; Moorchung et al., 2014). Hence, modulation of both these markers of inflammation by SBKT indicated its anti-inflammatory capabilities. Analysis of the NF-κB protein downstream expression of the pro-inflammatory cytokines IL-1β, IL-6, and TNF-α showed a modulation via SBKT treatment in the stimulated THP-1 cells. This finding holds importance since the onset of psoriasis disease involves the increased expression of NF-κB and release of the pro-inflammatory mediators and RNS (Kupper and Fuhlbrigge, 2004).

An anti-inflammatory activity study of the SBKT was performed using the λ-Carrageenan-stimulated Wistar rat inflammation model and the TPA-stimulated CD-1 mice psoriasis-like model. The TPA-stimulated CD-1 mice psoriasis-like model is well-established for studying the disease-modulating efficacy of test compounds (Madsen et al., 2016; Ma et al., 2018; Yang et al., 2018). Treatment of the Carrageenan-stimulated Wistar rats with a human equivalent dose of SBKT showed a significant decrease in the drug-induced paw volume increase and edema in the rats. Similarly, SBKT treatment at a human equivalent dose in the TPA-stimulated CD-1 mice revealed a modulation of the psoriasis-like inflammation and associated lesions in the mice ear. Reduction in the inflammatory lesions can be well correlated with the anti-inflammatory activity of the SBKT observed in the LPS-stimulated THP-1 cells, showing downregulation of inflammatory mediators.

Earlier studies have also shown the SBKT to possess anti-inflammatory properties through the modulation of pro-inflammatory cytokines, cyclooxygenase-2, inducible nitric oxide synthase, and inflammasome-associated IKK-β/NF-κB pathways (Jayashankar et al., 2012; Jayashankar et al., 2014; Suchal et al., 2016; Shi et al., 2017; Tanwar et al., 2018). The anti-inflammatory property of the SBKT can be related to the presence of fatty acids, such as polyunsaturated and omega-3 fatty acid components. These fatty acids have been reported to inhibit LPS-stimulated inflammation in inflammatory cells through modulation of Toll-like receptor 4, NF-κB, Nod-like receptor protein 3, cyclooxygenase-2, JAK, and P38 pathways and associated release of pro-inflammatory cytokines (Lee et al., 2003; Martínez-Micaelo et al., 2016; Hou et al., 2017). Hence, in our study, the presence of these fatty acids can be correlated with modulation of the NF-κB and pro-inflammatory cytokine inhibition observed in the stimulated THP-1 cells when treated with SBKT and inhibition of inflammation in the *in vivo* studies. Blocking of the TNF-α, the NF-κB pathways have been a focus of the anti-psoriasis treatments as it leads to the reduction in the inflammasome activation and downregulation of the cytokine such as IL-1β (Goldminz et al., 2013; Moorchung et al., 2014).

Traditionally, the SBKT plant has been called as the “wonder plant” for its therapeutic applications in several diseases. While our study demonstrated loss of cell viability in the THP-1 cells under *in vitro* conditions at higher doses. Similarly, using *in vivo* models, no toxicity has been reported for this plant’s part extracts and oils. Acute and sub-chronic toxicity studies performed in Wistar rats have shown no signs of toxicity and reported a no-observed-adverse-effect level of 10 ml/kg body weight (Zhao et al., 2017). Furthermore, no mutagenicity was observed from the SBKT exposure in histidine-dependent *Salmonella typhimurium* stain (Wen et al., 2018), suggesting no induction of genotoxicity by SBKT. Exposure to SBKT also did not induce any changes in sperm morphology and micronucleus formation rate in polychromatic erythrocytes obtained from mice orally treated with the oil (Wen et al., 2018). In the present study, we have not seen any change in the animal weights, food, or water consumption (data not shown). In a recently published article, clinical application of the SBKT extract in 10 psoriatic patients showed a significant reduction in their Psoriasis Area Severity Index scores and in Dermatology Life Quality Index scores within 4–8 weeks’ treatment, compared to placebo-treated patients who showed worsening signs in 4 weeks’ trial period (Boca et al., 2019). In another study, obese children aged 10–18 years treated with SBKT (800 mg/day) for 60 days were found with reduced levels of total cholesterol, triglyceride, leptin, fasting C peptide, oxidative stress, and carotid artery intima–media thickness, at the end of the treatment period (Virgolici et al., 2013). These clinical outcomes bode very well with the results reported here, suggesting an overall efficacy of SBKT in the treatment of psoriasis and general inflammations.

Besides having high nutritional and therapeutic values, SBKT can have other applications such as the development of lipid-based nano-drug-delivery vehicle as well as its incorporation into capsules, gelatine, and oral liquids as an emulsifier (Yang and Kallio, 2002). In our present study, it was observed that there is very little that SBKT, the wonder plant, cannot be used for. The present study adds a pharmacological body of evidence to its tradition-rich nutritional usage—natural nutraceutical—indeed.

## Conclusion

Finally, our study provided scientific evidence to the traditional wisdom that the SBKT obtained from the pulp of the seabuck thorn berries can be used as a therapeutic agent in subduing systemic inflammations and psoriasis-like lesions. Presence of high levels of saturated, monounsaturated, and polyunsaturated fatty acids along with other biomolecules in the oil significantly increases its values as a nutraceutical. In addition, the presence of high levels of clinically relevant lipids provides the opportunity to further explore the commercial and pharmaceutical applications of SBKT.

## Ethics Statement

The animal study protocol was approved by the Institutional Animal Ethical Committee of Patanjali Research Institute vide IAEC approval numbers: PRIAS/LAF/IAEC-008 and PRIAS/LAF/IAEC-022. All the experiments were performed in accordance with relevant guidelines and regulations.

## Author Contributions

AB provided a broad direction for the study, identified the test formulation, generated resources, and gave final approval for the manuscript. SS conducted the *in vivo* study, analyzed the data, and helped in manuscript writing and reviewing. KhJ assisted in animal handling and in performing *in vivo* studies. KaJ prepared the histopathological slides. RR performed the *in vitro* experiments. VS and KB performed data curing and wrote the manuscript. AV supervised overall research project planning, generated resources, and reviewed and finally approved the manuscript.

## Funding

This presented work has been conducted using research funds from Patanjali Research Foundation Trust, Haridwar, India.

## Conflict of Interest

The authors declare that the research was conducted in the absence of any commercial or financial relationships that could be construed as a potential conflict of interest.

## Abbreviations

SBKT, sea buckthorn oil; TNF-α, tumor necrosis factor-α; IL-6, interleukin-6; IL-1β, interleukin-1β; LPS, lipopolysaccharides; ELISA, enzyme-linked immunosorbent assay; RPMI, Roswell Park Memorial Institute 1640 Medium; FBS, fetal bovine serum; RNS, reactive nitrogen species; CPCSEA, Committee for the Purpose of Control and Supervision of Experiments on Animals; IAEC, Institutional Animal Ethical Committee; DEXA, dexamethasone; Na CMC, sodium carboxy methyl cellulose; ANOVA, analysis of variance; NC, normal control; TPA, 12-O-tetradecanoyl phorbol-13-acetate; VC, vehicle control; TPA CON, TPA control; INDO, indomethacin; T.A., topical application; SFA, saturated fatty acids; MUFA, monounsaturated fatty acids; PUFA, polyunsaturated fatty acids.
